# Functional and Radiological Outcomes of Extra-articular Distal Radius Fractures Treated With Percutaneous K-wiring Using the Ulna as a Cantilever: A Single-Centre Prospective Observational Study

**DOI:** 10.7759/cureus.115478

**Published:** 2026-08-31

**Authors:** Satyen P Joshi, Shubham v Shinde, Kishor Murkute, Nikhil Borde

**Affiliations:** 1 Orthopaedics, Dr. Vasantrao Pawar Medical College, Hospital & Research Centre, Nashik, IND; 2 Trauma, Arthroscopy and Joint Replacement, Lifeline Super Speciality Hospital, Solapur, IND; 3 Spine Surgery, Saarathi Spine and Orthopaedic Hospital, Parbhani, IND; 4 Orthopaedics, Borde Hospital, Akola, IND

**Keywords:** cantilever technique, distal radius fracture, functional outcome, k-wire fixation, percutaneous pinning

## Abstract

Background: Distal end radius fractures are among the most common orthopaedic injuries and can significantly impair wrist function if not managed appropriately. Various treatment modalities exist, but achieving stable fixation with minimal complications remains a challenge, especially in extra-articular fractures.

Aim: The aim of this study was to evaluate the functional outcome of distal end radius fractures treated by K-wiring using the ulna as a cantilever.

Methods: This prospective observational study included 80 skeletally mature patients with extra-articular distal end radius fractures treated by closed reduction and percutaneous K-wiring using the ulna as a cantilever. Patients were followed clinically and radiologically for three months. Functional outcomes were assessed using the Disabilities of the Arm, Shoulder and Hand (DASH) score, and radiological outcomes were assessed based on fracture union and time to radiological union during the follow-up period. The data were analysed using descriptive statistics. Categorical variables were presented as frequencies and percentages (N (%)), while continuous variables were expressed as mean ± standard deviation. DASH scores were analysed as a continuous measure and categorised as excellent, good, satisfactory, or poor according to the specified score ranges. No inferential statistical tests were performed.

Results: A total of 80 patients were included in the study. The mean age was 52.2 years, with the majority of patients (n = 32, 40%) belonging to the 41-60-year age group. Female patients predominated, accounting for 48 (60%) of the study population. Road traffic accidents were the most common mode of injury, occurring in 45 (56.3%) patients, followed by falls in 35 (43.7%) patients. Radiological union was achieved within 8-12 weeks in 36 (45%) patients, while non-union was observed in 3 (3.8%) patients. Functional outcomes were excellent in 21 (26.3%) patients, good in 38 (47.5%) patients, satisfactory in 18 (22.5%) patients, and poor in 3 (3.7%) patients. Overall, good to excellent outcomes were achieved in 59 (73.8%) patients.

Conclusion: Percutaneous K-wiring using the ulna as a cantilever appears to be a feasible treatment option for appropriately selected extra-articular distal radius fractures and was associated with acceptable short-term functional and radiological outcomes in this prospective observational study.

## Introduction

Distal end radius fractures are among the most common fractures encountered in orthopaedic practice, accounting for a significant proportion of upper limb injuries across all age groups. They are particularly prevalent in elderly individuals due to osteoporosis and in younger populations following high-energy trauma such as road traffic accidents [1]. These fractures can lead to considerable functional impairment if not managed appropriately, affecting wrist mobility, grip strength, and overall hand function.

The management of distal radius fractures has evolved over time, ranging from conservative treatment with casting to various surgical interventions aimed at achieving anatomical reduction and stable fixation. The primary goal of treatment is to restore the normal anatomy of the wrist joint, maintain alignment, and allow early mobilisation to achieve optimal functional outcomes [2]. Unstable fractures, in particular, often require surgical fixation to prevent loss of reduction and long-term complications such as malunion, stiffness, and post-traumatic arthritis [3].

Closed reduction and percutaneous K-wire fixation is a widely used and minimally invasive technique for the treatment of extra-articular distal radius fractures. It provides adequate stability while preserving soft tissue integrity and minimising surgical morbidity [4]. However, maintaining reduction in unstable fractures remains a challenge, especially in cases with comminution or poor bone quality [5].

The concept of using the ulna as a cantilever in K-wire fixation offers a biomechanically advantageous method for stabilising distal radius fractures. In this technique, the intact ulna acts as a supportive structure, allowing trans-ulnar K-wires to function as a cantilever system that helps maintain fracture reduction and alignment [6]. This method provides additional stability compared to conventional pinning techniques and may reduce the risk of displacement during the healing period.

Several studies have emphasised the importance of stable fixation techniques in improving functional outcomes in distal radius fractures. Percutaneous pinning methods have been shown to provide satisfactory results with minimal complications when proper technique and patient selection are ensured [7]. Additionally, radiological parameters such as radial height, radial inclination, and volar tilt play a crucial role in determining long-term functional outcomes, highlighting the need for techniques that can effectively maintain these parameters [8].

The use of K-wire fixation is also associated with advantages such as shorter operative time, reduced cost, and minimal soft tissue dissection, making it a preferred option in many settings, especially in resource-limited environments [9]. However, complications such as pin tract infection, loosening of wires, and loss of reduction can still occur, necessitating careful surgical technique and postoperative management [10].

Despite various available treatment modalities, there is still ongoing debate regarding the most effective method for achieving optimal functional and radiological outcomes in distal radius fractures. The cantilever technique using the ulna as an internal support offers a promising alternative that combines simplicity with biomechanical stability.

The present study aimed to evaluate the functional and radiological outcomes of extra-articular distal radius fractures treated with closed reduction and percutaneous K-wiring using the ulna as a cantilever. The secondary objective was to assess fracture union and postoperative complications during the follow-up period. The justification for this study lies in the need for a simple, cost-effective, and minimally invasive technique that provides stable fixation and good functional results, especially in extra-articular fractures of the distal radius. Conventional methods may be associated with complications such as loss of reduction, prolonged immobilisation, or higher surgical morbidity, thereby necessitating evaluation of alternative techniques like the cantilever method. The findings of this study are expected to provide valuable insights into the clinical utility of this technique and its role in routine orthopaedic practice. In the future, the outcomes of this study may contribute to establishing standardised treatment protocols, encouraging wider adoption of this technique in suitable cases, and promoting further research through larger multicentre studies to validate its long-term efficacy and safety.

## Materials and methods

This prospective observational study was conducted in the Department of Orthopaedics at Dr. Vasantrao Pawar Medical College and Hospital between May 2021 and December 2022. Institutional Ethics Committee approval was obtained on 27 May 2021 prior to commencement of patient recruitment and data collection.

A total of 80 skeletally mature patients with extra-articular distal radius fractures who met the predefined inclusion and exclusion criteria were included in the study. No formal power analysis was performed for comparative hypothesis testing because this was a single-arm prospective observational study with descriptive analysis. The study was designed as a prospective, non-comparative study to evaluate the clinical and radiological outcomes of the cantilever K-wire technique in extra-articular distal radius fractures. A comparative group was not included, as the primary objective was to assess the functional and radiological outcomes and feasibility of this specific technique. Accordingly, the study was not designed to determine the superiority of the cantilever technique over other fixation methods such as volar plating or conventional K-wire fixation.

Patients aged more than 18 years with extra-articular distal radius fractures presenting within two weeks of injury and planned for surgical management with percutaneous K-wire fixation using the ulna as a cantilever were included in the study. Patients with intra-articular distal radius fractures, associated distal ulna fractures, neurovascular compromise, ipsilateral proximal forearm fractures, and paediatric fractures with open physes were excluded from the study.

All patients presenting to the emergency department or outpatient department were admitted and evaluated thoroughly. A detailed history was obtained regarding the mode of injury, duration since trauma, and associated comorbidities, along with a thorough clinical examination of the affected limb. Radiological evaluation was performed using anteroposterior and lateral X-rays of the wrist joint to confirm the diagnosis and assess fracture characteristics. Preoperative anteroposterior and lateral radiographs were obtained to assess the fracture configuration (Figure 1).

**Figure 1:**
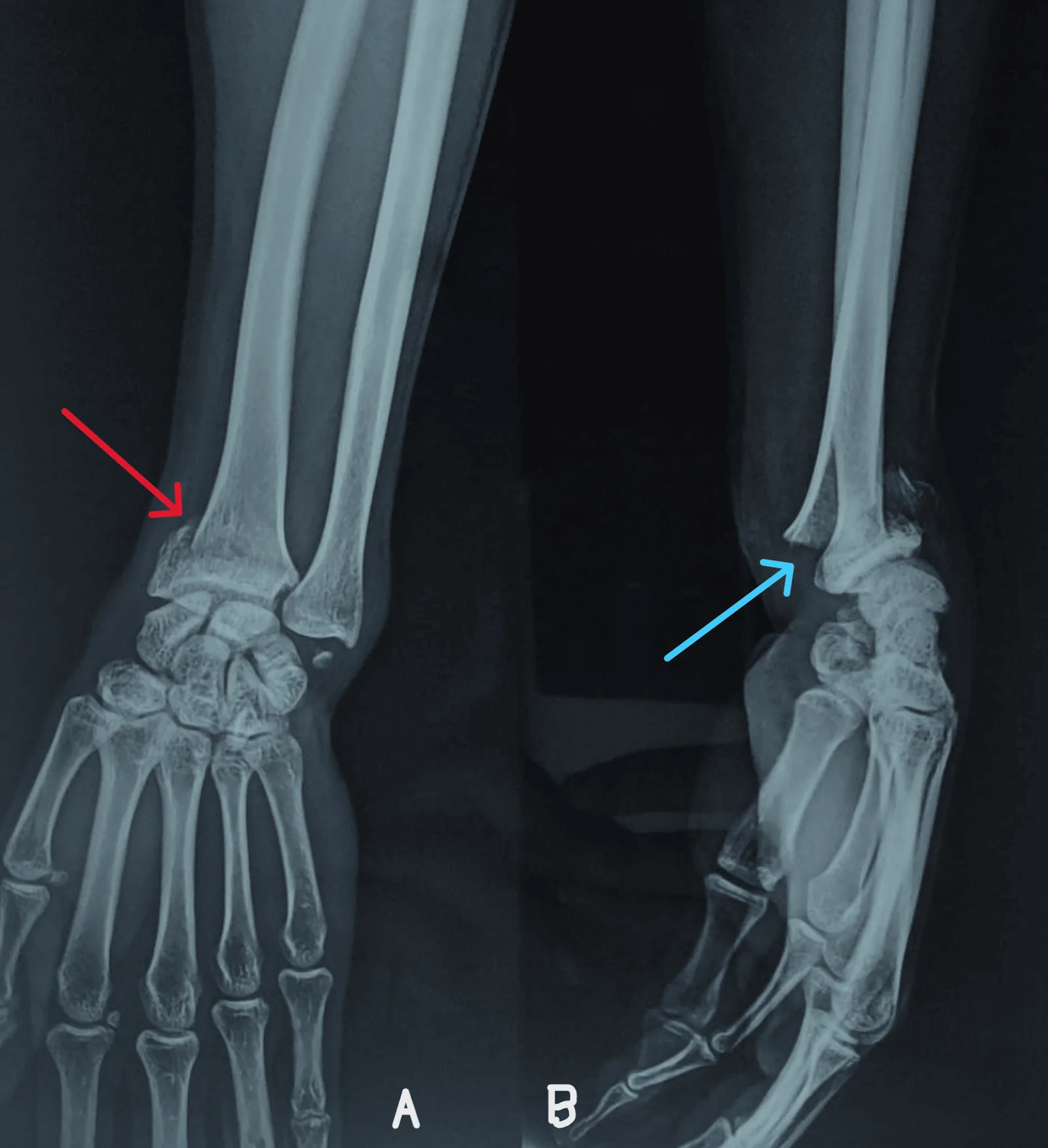
Preoperative radiographs of extra-articular distal radius fracture. (A) Anteroposterior radiograph demonstrating the distal radius fracture (red arrow). (B) Lateral radiograph demonstrating dorsal displacement of the distal radius fracture (blue arrow).

Preoperatively, the affected limb was immobilised in an above-elbow slab and elevated to reduce swelling and pain. All routine preoperative investigations, including haemoglobin, blood sugar, blood urea, platelet count, and electrocardiogram (when indicated), were performed, and anaesthetic fitness was obtained prior to surgery.

Surgical steps

The patient was placed supine on the operating table. The patient was positioned using the hanging-hand technique under fluoroscopic guidance, and the C-arm was positioned to obtain anteroposterior and lateral views throughout the procedure (Figure 2). Manipulation was performed, and the fracture was reduced and checked with an image intensifier. The affected arm was then suspended from an IV stand with the help of cotton bandages tied to the index and middle fingers. This also maintained gravity-assisted sustained traction. The final position was such that the shoulder was in 90° of abduction and external rotation, the elbow was in 90° of flexion, and the forearm was in the mid-prone position. The image intensifier was adjusted to this final limb position such that it offered an unhindered anteroposterior view of the wrist joint. The limb was then prepared, painted, and draped, taking all aseptic precautions. Two 1.8-mm K-wires were passed from the ulnar head into the distal radius fracture fragment, crossing the distal radioulnar joint parallel to the radiocarpal joint. These K-wires were inserted in such a way that they crossed each other when an end-on view of the wrist was obtained. Another 1.8-mm K-wire was inserted from the radial styloid in an oblique direction, crossing the fracture line and taking purchase of the opposite radial cortex. Immediate postoperative radiographs demonstrated satisfactory fracture reduction and K-wire fixation with below-elbow cast immobilisation (Figure 3). Care was taken not to injure the superficial branch of the radial nerve or the tendons of the first and third extensor compartments. A below-elbow cast was applied after sterile dressing.

**Figure 2:**
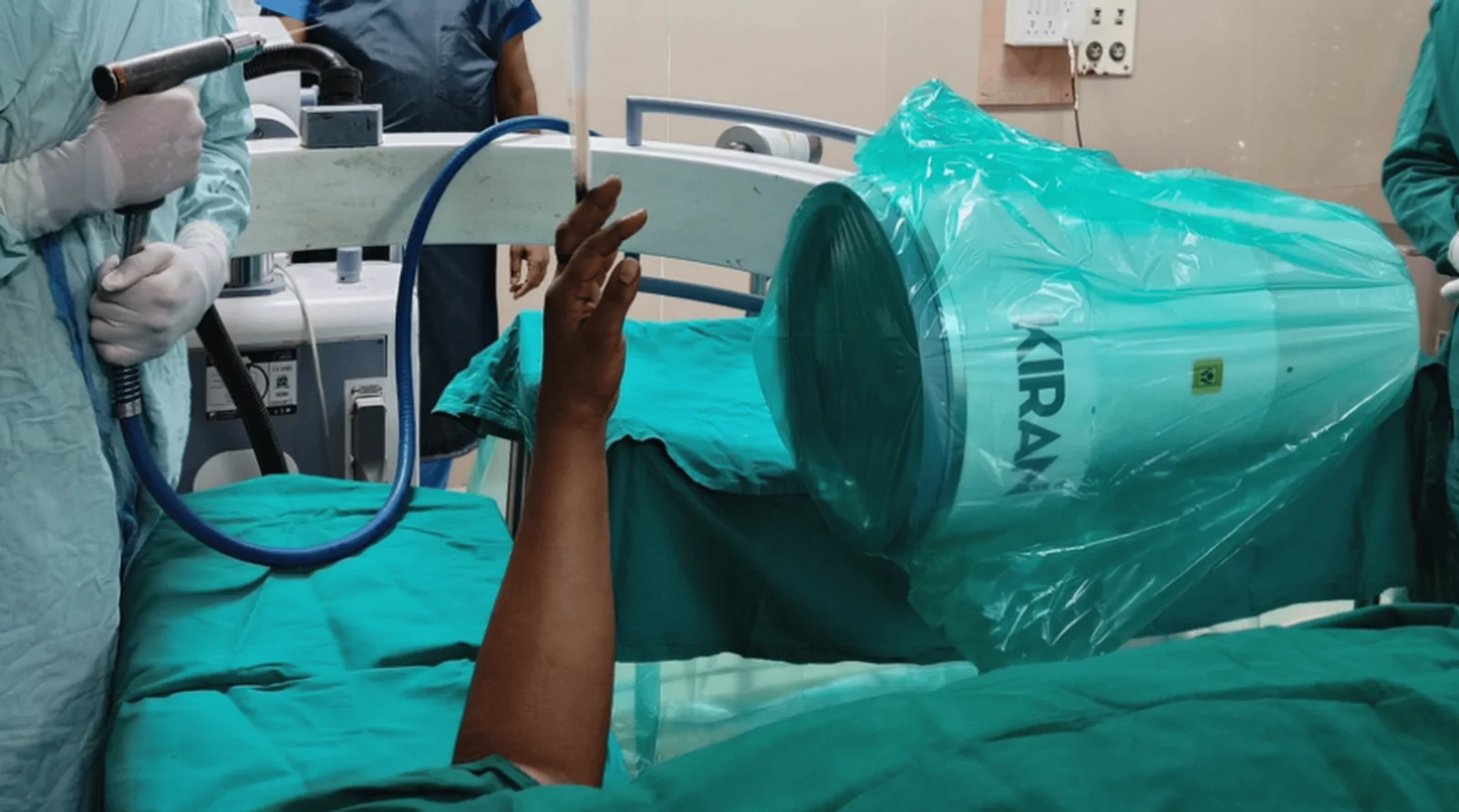
Intraoperative positioning and C-arm setup. Intraoperative photograph demonstrating patient arm positioning in suspended (“hanging hand”) position along with C-arm positioning during percutaneous K-wire fixation using the ulna as a cantilever technique.

**Figure 3:**
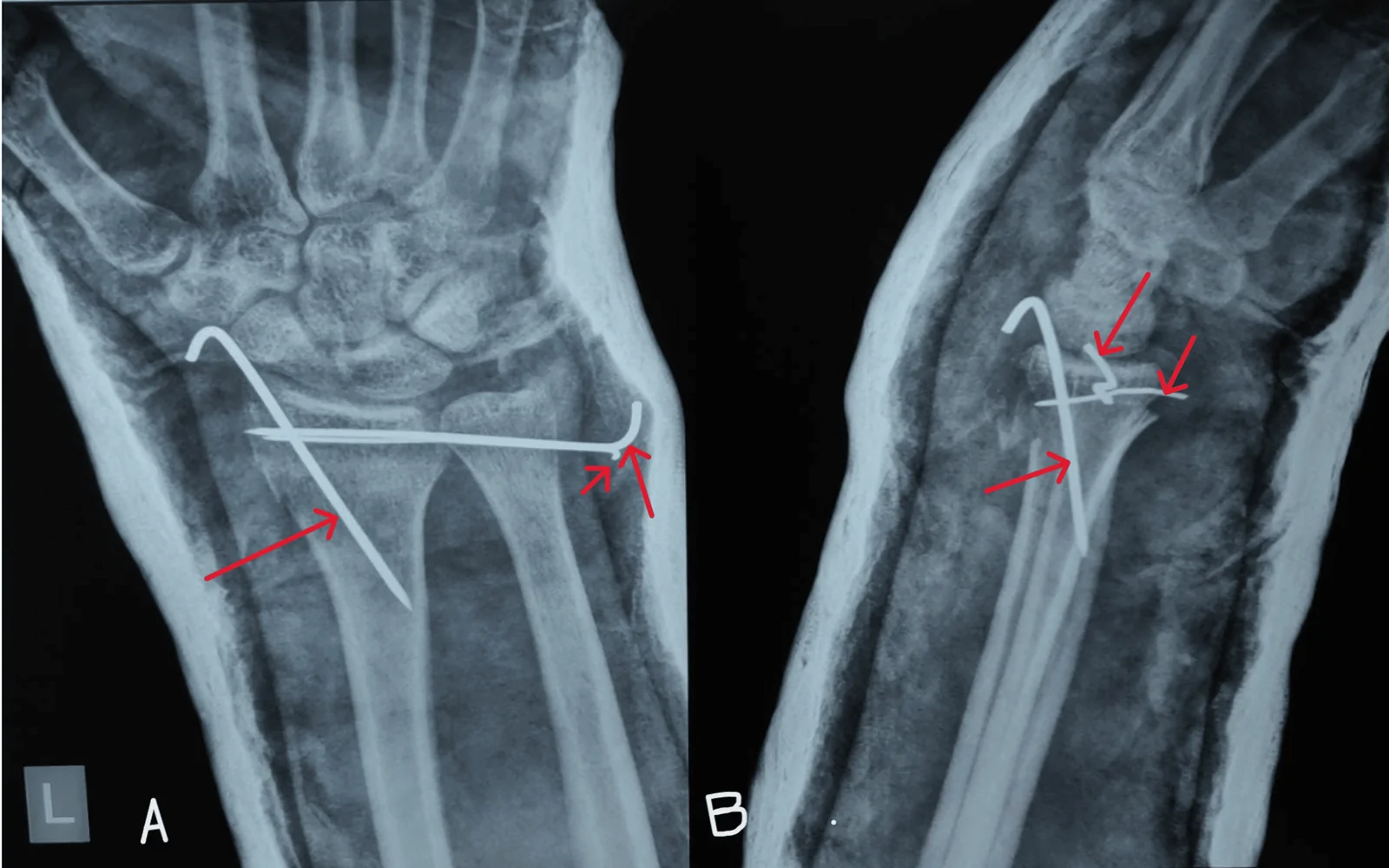
Immediate postoperative radiographs following closed reduction and percutaneous K-wire fixation using the ulna as a cantilever. (A) Anteroposterior radiograph demonstrating satisfactory fracture reduction and stabilisation with trans-ulnar K-wire fixation (red arrows). (B) Lateral radiograph demonstrating maintenance of fracture alignment with K-wire fixation and below-elbow cast immobilisation (red arrows).

For postoperative care and rehabilitation, patients were immobilised in a below-elbow cast in ulnar deviation with slight wrist flexion for six weeks, with active mobilisation of the fingers and elbow encouraged during this period. Gradual wrist mobilisation was initiated after cast removal, and patients were encouraged to perform mobilisation exercises throughout the follow-up period. Heavy lifting was restricted for 12-16 weeks, after which patients were allowed to resume heavy activities at approximately three months.

Patients were followed clinically and radiologically at regular intervals at three weeks, six weeks, and three months. Follow-up radiographs demonstrated maintained fracture reduction and progressive radiological union (Figure 4). Functional outcomes were assessed using the Disabilities of the Arm, Shoulder and Hand (DASH) score at 3 months, with scores ranging from 0 to 100, where lower scores indicate better upper-limb function. Radiological outcomes were assessed based on fracture union and the time to radiological union during follow-up. For descriptive categorisation of functional outcomes, DASH scores of 0-5 were considered excellent, 6-15 as good, 16-35 as satisfactory, and >35 as poor.

**Figure 4:**
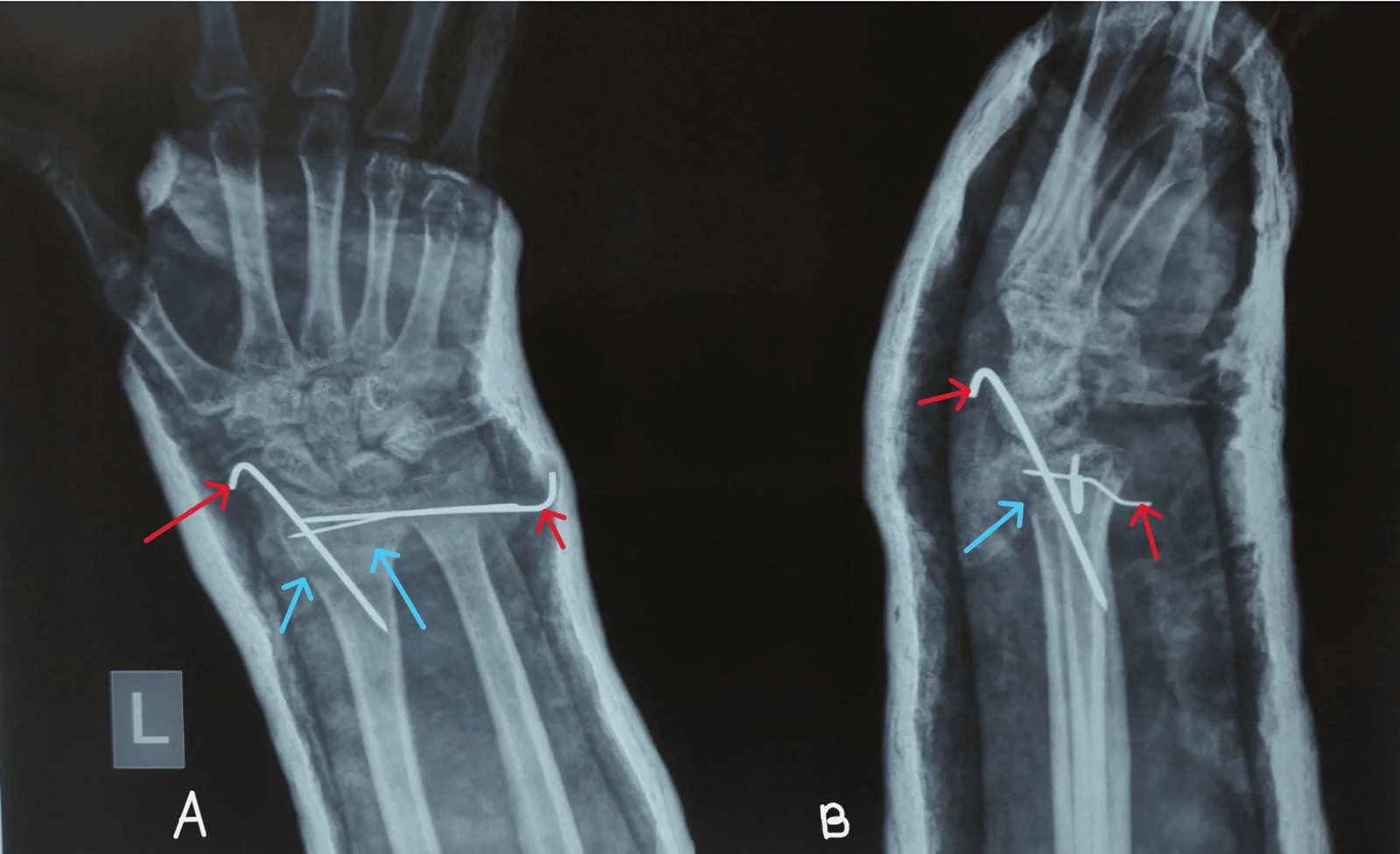
Follow-up radiographs demonstrating progression of fracture healing after K-wire fixation. (A) Anteroposterior radiograph showing maintained reduction, K-wires (red arrows) and evidence of fracture union at the fracture site (blue arrow). (B) Lateral radiograph demonstrating satisfactory alignment, K-wires (red arrows) and progressive radiological union of the distal radius fracture (blue arrow).

Statistical analysis

The collected data were analysed using descriptive statistics. Categorical variables were summarised as frequencies and percentages (N (%)), while continuous variables were expressed as mean and standard deviation. The DASH score was analysed as a continuous functional outcome measure and was additionally categorised into excellent, good, satisfactory, and poor outcomes according to the score ranges described in the Methods section. As this was a prospective observational study without a comparative control group, no inferential statistical tests were performed. The final sample comprised 80 patients. This study was reported in accordance with the Strengthening the Reporting of Observational Studies in Epidemiology (STROBE) guidelines. 

## Results

A total of 80 patients were included in this study. The age of the patients ranged from 21 to 80 years, with a mean age of 52.2 years. The majority of patients belonged to the 41-60-year age group, accounting for 32 (40%) patients, followed by the 21-40-year and 61-80-year age groups, each comprising 24 (30%) patients. Females predominated, comprising 48 (60%) patients, while males accounted for 32 (40%) patients.

Left-sided injuries were slightly more common, occurring in 43 (53.7%) patients, compared to right-sided injuries in 37 (46.3%) patients. The most common mode of injury was a road traffic accident, seen in 45 (56.3%) patients, followed by a fall in 35 (43.7%) patients (Table 1). 

**Table 1:** Baseline demographic and injury characteristics of the study population (N = 80).

Characteristic	n (%)
Age, years	
21-40	24 (30.0)
41-60	32 (40.0)
61-80	24 (30.0)
Mean age, years	52.2
Sex	
Female	48 (60)
Male	32 (40)
Mode of injury	
Road traffic accident	45 (56.3)
Fall	35 (43.7)

Radiological union was most commonly achieved between 8 and 12 weeks in 36 (45%) patients, followed by 12 to 16 weeks in 21 (26.3%) patients and within 6 to 8 weeks in 15 (18.8%) patients. Delayed union beyond 16 weeks occurred in 5 (6.3%) patients, while non-union was observed in 3 (3.8%) patients, indicating a high overall union rate (Table 2). 

**Table 2:** Time to radiological union

Time to radiological union	Patients, n (%)
Within 6-8 weeks	15 (18.8)
8-12 weeks	36 (45.0)
12-16 weeks	21 (26.3)
Beyond 16 weeks	5 (6.3)
Non-union	3 (3.8)

At the three-month follow-up, functional outcome assessment demonstrated good results in 38 (47.5%) patients and excellent results in 21 (26.3%) patients, while satisfactory outcomes were observed in 18 (22.5%) patients and poor outcomes in 3 (3.8%) patients. Overall, good to excellent outcomes were achieved in 59 (73.8%) patients, indicating favourable functional recovery with this technique. The mean DASH score at three months was 11.24 ± 6.02, with a range of 2.7-36.7.

**Table 3:** Functional outcomes of the study population DASH: Disabilities of the Arm, Shoulder and Hand.

Functional outcome	n (%)
Excellent	21 (26.3)
Good	38 (47.5)
Satisfactory	18 (22.5)
Poor	3 (3.8)
Good to excellent	59 (73.8)
DASH score, mean ± SD	11.24 ± 6.02
DASH score range	2.7–36.7

## Discussion

The present prospective study evaluated the functional and radiological outcomes of distal end radius fractures treated by closed reduction and percutaneous K-wiring using the ulna as a cantilever in 80 patients. The technique is based on a simple biomechanical principle where the intact ulna acts as a stable support, allowing trans-ulnar K-wires to function as a cantilever to maintain fracture reduction and alignment.

In the current study, the mean age was 52.2 years, with the majority of patients belonging to the 41-60-year age group, comprising 32 (40%) patients, followed by the 21-40-year and 61-80-year age groups, each comprising 24 (30%) patients. This age distribution is consistent with the epidemiology of distal radius fractures, where middle-aged and elderly individuals are more commonly affected due to osteoporotic bone changes and increased susceptibility to trauma. Similar observations have been reported by Venkatesh et al., who noted a higher incidence of distal radius fractures in the adult and elderly population undergoing K-wire fixation [11].

A female predominance was observed in the present study, with 48 (60%) female patients and 32 (40%) male patients. This finding aligns with existing literature. The higher incidence in females is commonly attributed to postmenopausal osteoporosis and low bone mineral density, making them more prone to fractures even with low-energy trauma.

In terms of mode of injury, road traffic accidents were the most common cause, accounting for 45 (56.3%) patients, followed by falls in 35 (43.7%) patients. This finding reflects a mixed pattern of high-energy and low-energy trauma. While Western studies often report falls as the predominant cause, Indian studies frequently highlight road traffic accidents as a leading mechanism due to higher exposure to vehicular trauma. Thomas et al. reported similar findings, with both road traffic accidents and falls being major contributors [12].

Radiological union was achieved within 8-12 weeks in 36 (45%) patients, followed by 12-16 weeks in 21 (26.3%) patients and within 6-8 weeks in 15 (18.8%) patients. Delayed union beyond 16 weeks occurred in 5 (6.3%) patients, while non-union was observed in only 3 (3.8%) patients, indicating a high overall union rate. These findings are comparable with studies by Patil et al., who demonstrated that percutaneous K-wire fixation provides effective fracture healing and maintains reduction in extra-articular distal radius fractures [13]. Chaudhary et al. also reported early union with stable fixation techniques, emphasising the importance of maintaining anatomical alignment [14]. The slightly longer union time in some patients may be attributed to factors such as age, bone quality, and compliance with immobilisation.

Functional outcomes in this study were encouraging, with excellent results observed in 21 (26.3%) patients, good results in 38 (47.5%) patients, satisfactory results in 18 (22.5%) patients, and poor results in only 3 (3.7%) patients. Overall, good to excellent outcomes were achieved in 59 (73.8%) patients [15,16]. These findings are comparable with previously published studies. The favourable outcomes in the present study can be attributed to adequate fracture stabilisation, maintenance of alignment, and early initiation of mobilisation following cast removal.

The cantilever principle used in this technique provides an added biomechanical advantage. By utilising the ulna as a stable support, the trans-ulnar K-wires help prevent collapse of the distal radius fragment and maintain reduction throughout the healing period. Jagtap et al. described similar techniques and highlighted their effectiveness in maintaining fracture alignment with minimal soft tissue disruption [15]. The cantilever configuration is intended to provide additional mechanical support for maintaining fracture alignment, particularly in appropriately selected extra-articular fracture patterns. However, the present study was not designed to directly compare its stability with other fixation methods.

Another important aspect of this technique is its minimally invasive nature, which reduces surgical morbidity, operative time, and cost. Unlike volar plating, which requires extensive soft tissue dissection, K-wire fixation preserves soft tissues and blood supply, thereby promoting fracture healing. This makes it especially suitable for use in resource-limited settings where cost and infrastructure are important considerations.

Complication rates in the present study were low, with non-union occurring in 3 (3.8%) patients and stiffness in 16 (20%) patients, indicating that the technique is relatively safe. Common complications associated with K-wire fixation, such as pin tract infection or wire loosening, occurred in 2 (2.5%) patients and were minimal and manageable. The low complication rate further supports the reliability of this technique.

Overall, the findings of this study demonstrate that K-wiring using the ulna as a cantilever is an effective and practical technique for managing extra-articular distal radius fractures. It provides satisfactory radiological union, good functional recovery, and minimal complications. The technique combines percutaneous fixation with a relatively simple construct and may therefore represent a practical option in appropriately selected patients. Comparative studies are required to determine its relative effectiveness and cost-effectiveness compared with other fixation methods.

Limitations

The present study has certain limitations. It was conducted at a single centre with a relatively limited sample size of 80 patients, which may affect the generalisability of the findings. The follow-up period was limited to three months. Therefore, the present study evaluates short-term functional and radiological outcomes and cannot assess long-term functional recovery, late loss of reduction, post-traumatic arthritis, or delayed complications. Longer follow-up studies are required to evaluate these outcomes. Additionally, the absence of a comparative control group treated with alternative fixation methods limits direct comparison of the effectiveness of the cantilever K-wire technique with other established treatment modalities. Further multicentre randomised studies with larger sample sizes and longer follow-up are required to validate these findings.

## Conclusions

Closed reduction and percutaneous K-wiring using the ulna as a cantilever appears to be a feasible treatment option for appropriately selected extra-articular distal radius fractures and was associated with acceptable short-term radiological and functional outcomes in this prospective observational study. The technique may be considered in selected patients; however, comparative studies with larger sample sizes and longer follow-up are required to determine its relative effectiveness compared with other fixation methods.
